# *Panax notoginseng* flower extract ameliorates chronic unpredictable mild stress -induced depression-like behaviors in mice by reducing neuroinflammation

**DOI:** 10.22038/ajp.2025.26164

**Published:** 2026

**Authors:** Feiya Zhu, Jiayu Xie, Yang Zhao, Yiting Wang, MG Matsabisa, Minke Tang

**Affiliations:** 1 *School of Chinese Materia Medica, * *Beijing* *University* *of Chinese Medicine, Beijing, 100029, China.*; 2 *Beijing Institute for Chinese Medicine Research, Beijing University of Chinese Medicine, Beijing, 100029, China.*; 3 *Faculty of Health Sciences, University of the Free State, Bloemfontein 9300, South Africa.*

**Keywords:** Panax notoginseng flower Ginsenosides, Depression, Chronic mild unpredictable stress, Astrocytes, Neuroinflammation

## Abstract

**Objective:**

To evaluate whether *Panax notoginseng* flower extract (PNF) can alleviate depression-like behavior caused by chronic unpredictable mild stress (CUMS) in mice and to explore its relations to neuroinflammation.

**Materials and Methods:**

C57BL/6J mice were subjected to CUMS for 7 weeks to induce depressive-like behaviors. Then PNF 1.7 or 3.4 g/kg was administered via intragastric gavage once a day for 4 consecutive weeks. After behavioral assessment, the systemic inflammation and neuroinflammation were investigated by detecting inflammatory factors in serum and brain with enzyme-linked immunosorbent assay (ELISA). The serum levels of adrenocorticotropic hormone (ACTH) and glucocorticoids (GC) were also determined. The activation of microglia and astrocyte was investigated by immunohistochemistry. The chemical components in PNF were analyzed with Ultra-high performance liquid chromatography/MS (UPLC/MS).

**Results:**

PNF 1.7 and 3.4 g/kg treatment alleviated depressive behavior in CUMS mice in various behavioral studies. In both serum and brain, PNF treatment significantly counteracted the CUMS-induced enhancement of typical pro-inflammatory factors, Tumor Necrosis Factor alpha (TNF-α), Interleukin-1β (IL-1β), and IL-6 and counteracted the CUMS-induced decrease of anti-inflammatory factorIL-10. Treatment with PNF also attenuated the CUMS-induced serum ACTH and GC elevation. Immunohistochemical analysis revealed that PNF treatment significantly reduced the number of ionized calcium-binding adapter molecule 1 (Iba-1) and glial fibrillary acidic protein (GFAP) positive cells in the brains of CUMS mice, indicating an inhibition of microglial and astrocytic activation. UPLC/MS study suggest that ginsenoside Rh1 is the main ginsenoside in the extract.

**Conclusion:**

PNF ameliorates CUMS-induced depression-like behaviors in mice, which may be mainly related to reducing neuroinflammation in the brain.

## Introduction

According to the World Health Organization, about 280 individuals worldwide experience depression, and 700,000 people die of depression every year (Ma et al. 2020). The situation has been exacerbated by three years of Coronavirus Disease 2019 (COVID-19), and depression has become a major illness that seriously affects human health (Ma et al. 2020). Depression frequently correlates with anxiety disorders and more pronounced cognitive impairments (Beblo et al. 2011), severely impairing the psychological and social functioning of patients. Since the 1950s, monoamine oxidase inhibitors and monoamine neurotransmitter reuptake inhibitors have been successively employed for depression treatment, many depressed patients have been benefited from the monoamine related intervention (López-Muñoz and Alamo 2009). However, the clinical data accumulated in the past half century show that the long-term intervention of monoamine neurotransmitters brings numerous dreadful adverse effects (Blackwell 1981). What's more, about one-third of the depressed patients show no improvement to monoamine neurotransmitter-related antidepressants drugs (Ruhé et al. 2012; Stewart et al. 2014). Given these limitations, it is imperative to discover new targets and develop novel therapeutic drugs against depression threat (Borbély et al. 2022; Tsugiyama et al. 2023). 

Neuroinflammation in the central nervous system (CNS) refers to an immune-driven reaction occurring in the brain or spinal cord, primarily triggered by the release of signaling molecules like cytokines and chemokines, alongside reactive oxygen species (ROS) and various secondary messengers (Ahmad et al. 2022). Neuroinflammation is thought to be a physiological attempt to eliminate injurious stimuli within the CNS and initiate healing processes to protect CNS cells and its overall function. However, an excessive inflammatory response can be harmful. Extensive research indicates that neuroinflammation is strongly linked to diverse neurological disorders (Sarno et al. 2021), such as multiple sclerosis (Rosenberg 2002), Alzheimer's disease (Thakur et al. 2023), and Parkinson's disease (Isik et al. 2023), etc. Maes et al (Maes et al. 1995) and Levine et al (Levine et al. 1999) reported in 1995 and 1999, respectively, that depression was associated with inflammatory makers in the blood and cerebrospinal fluid, and subsequent researchers have found that excessive immune stimulation can elicit depressive-like behaviors both in animals and humans (Zhao et al. 2019). Subsequent studies increasingly implicate neuroinflammation in depressive disorders (Ratajczak et al. 2019; Carvalho et al. 2010). It is therefore becoming a growing interest to improve depression by reducing excessive neuroinflammatory response. Numerous plants have demonstrated antidepressant properties in traditional medicine (Amin et al. 2015; Sadeghi et al. 2023), and there may be potential for discovering new advancements through these plants.

*Panax notoginseng* flower, a perennial herb of the Araliaceae family, which is mainly produced in Yunnan and Sichuan in China. Studies have shown that *P. notoginseng* flowers exhibit notable neuroprotective properties. Saponins extracted from this plant have been found to enhance hematoma absorption during the initial stages of hemorrhagic stroke in rats (Nie et al. 2006) and to shield neurons from damage while aiding functional recovery in patients following cerebral hemorrhage (Wei et al. 2007). Moreover, combining icariin with these saponins has proven effective in both preventing and treating Alzheimer's disease in rat models. This combination also alleviates learning and memory impairments and reduces blood viscosity by safeguarding neurons from oxidative stress in ischemic brains (Zheng et al. 2008). Beyond saponins, a flavanol glycoside known as Radix Notoginseng flavonol glycoside (RNFG), extracted from *P. notoginseng*, has demonstrated neuroprotective effects against amyloid-β-induced cell death and toxicity at the cellular level. Additionally, it enhances learning and memory processes in rats (Choi et al. 2010). *Panax notoginseng* flower contain high levels of ginsenosides (Zhang et al. 2018), which has been extensively demonstrated with significant anti-inflammatory effects (Liu et al. 2023). Numerous studies have shown that stressors such as major life events, are critical proximal risk factors for major depressive disorders (MDDs) (Monroe et al., 2007). It is well established that inflammation is a major adverse consequence of stress, as evidenced by changes at the protein level (e.g. pro-inflammatory cytokines), intracellular signaling pathways (e.g. transcription factors), and whole-genome expression (e.g. gene programming and transcriptional alterations) (Slavich and Irwin, 2014). Therefore, this study used chronic unpredictable mild stress (CUMS) to elicit depression-related behaviors in mice and investigate the effect of *P. notoginseng* flower on depressed mice. The results showed that Panax ginseng flower extract (PNF) could significantly alleviate CUMS induced depression-like behaviors in mice, which may be attributed to reducing the neuroinflammation.

## Materials and Methods

### Ethical approval

All animal research adhered to the National Institutes of Health’ s Guidelines for laboratory animal care. The experimental procedures received approval from the Ethics Committee at Beijing University of Chinese Medicine (BUCM-2021082802-3001). We took every possible measure to reduce animal suffering and to decrease the study's animal usage.

### Animals

C57BL/6J mice (n=100 with half male and half female), weighing 18-22 g, were acquired from SPF (Beijing) Biotechnology (license: SCXK [Jing] 2019-0010, Beijing, China). The mice were maintained in a controlled environment at 23±1°C with humidity levels between 30% and 70%. They experienced a 12-hr light/dark cycle, with lights activating at 8 AM, and had free access to lab food and water. To minimize differences caused by rhythms, all animals were sourced from the same batch. During the experiment, animals of different sexes were housed separately and were not allowed to interact.

### Drugs and Reagents

*Panax notoginseng* flower was obtained from Xinju Yiguang Health Science (An Guo, China), and it was extracted with 50% alcohol. After recovery of ethanol solvent by decompression distillation at 40°C, the extract was adjusted to 0.34 g/ml. The Enzyme-linked immunosorbent assay (ELISA) kits, including tumor necrosis factor-alpha (TNF-α, KT2132-A), Interleukin-1 beta (IL-1β, KT2040A), Interleukin-6 (IL-6, KT2163A), Interleukin-10 (IL-10, KT2176A), adrenocorticotropic hormone (ACTH, KT2554A), and corticosterone (GC, KT2854A), were all purchased from Jiangsu Keter Biological Technology Co., Ltd. (Chang Zhou, China). Primary antibodies, including ionized calcium binding adaptor molecule-1 (Iba1, ab178846) and glial fibrillary acidic protein (GFAP, ab7260) were purchased from Abcam (MA, USA). The secondary antibodies were purchased from Zhongshan Jinqiao Biotechnology (Beijing, China).

### Chronic Unpredictable Mild Stress (CUMS)

After an acclimation period of 7 days, CUMS protocols were applied to elicit depressive-like behaviors, following Mizuki's method with slight adjustments (Sharma et al. 2024). Briefly, the unpredictable mild stress are water and food deprivation for 24 hr, cage tilted at 45°C day/night reversal, damp bedding, restraint, tail pinching for 1 min, intense flashing eyes, ultrasonic noise, 4℃ water, etc., and more than two stimulation modalities were given every day. It was ensured that the same stress modality was not repeated within three days to prevent the mice from being able to anticipate the upcoming stimuli in advance, which would have effects on the modeling. After 7 weeks’ stress stimulation, the CUMS mice were randomly divided into 4 groups, CUMS, CUMS+Fluoxetine 10 mg/kg, CUMS+PNF 1.7 g/kg, and CUMS+PNF 3.4 g/kg, 20 mice in each group with half male and half female. Fluoxetine or PNF was administrated orally via intragastric gavage once a day consecutively for 4 weeks. A control group of 20 mice with half male and half female without CUMS stimulation was set up at the beginning. The mice in control group received same volume of water via intragastric gavage when the Fluoxetine or PNF treatment started for CUMS mice.

### Sucrose Preference Test

The sucrose preference test evaluated anhedonia-like behavior (Primo et al. 2023). The test was conducted after 4-weeks fluoxetine or PNF. The mice were singularly placed in a box with two bottles of 1% fructose before being denied food and water entry for 24 hr before the exam. One of the 1% sugar bottle was replaced with a water-filled one after 24 hr. The process was continued for another 24 hr, during which the sucrose bottle and the water bottle swapped places every 12 hr. Following the above-mentioned training, the tests were carried out in individual cage with a water jug and 1% sucrose for two hours. The bottles swapped places every hour. The sucrose preference was determined by the ratio of fructose use size to the overall amount of sucrose and water consumed.

### Forced Swimming Test (FST)

Generally, rodent depressive-like behavior can be assessed using the forced swimming test (FST) (Porsolt et al. 1977). At 25°C, each mouse was immersed alone in a clear glass jug with a 15 cm depth. The Etho-Vision XT9 technique was used to analyze the final four minutes of the mice's six-minute swim.

### Tail Suspension Test (TST)

The tail suspension test has become a well-known model for evaluating mice antidepressant-like effects since its creation nearly 20 years ago (Cryan et al. 2005). At the TST frame's top, mice were adhered 1 cm from the tail idea. Immobility was measured in the last 4 minutes of a 6-minute observation.

### Open field test (OFT)

The open-field test is used in research on the neurobiological foundations of anxiety and to identify novel anxiolytic agents and drug targets (Kraeuter et al. 2019a). Each OFT unit features an opaque plastic box measuring 44 × 44 × 44 cm, topped with a video recording device. The mice were given a 10-minute adaptation time in the OFT before the test, followed by a 5-minute test the following time. The mice were fasted for 24 hr before test. During test, a food ball with a diameter of 3 cm was placed in the center of each field. The time when the mice first accessing the food was noted as the latency period. If the mice fail to reach the food within 5 minutes, then the latency period was recorded as 5 minutes.

### Elevated Plus Maze Test (EPM)

The elevated plus maze assesses anxiety-related responses in rodents (Kraeuter et al. 2019b). The Elevated Plus Maze features four arms, each 66 cm long and 5 cm wide, arranged in a cross pattern with similar arms facing one another. It consists of two open arms and two enclosed arms and is raised 50 cm off the ground. The experiment began by positioning the mice at the intersection's center. A video recording setup was installed above the maze to record the movement trace. The ratio of the time that the mice spend in the open arm, and the times the mice enter the open arms are counted.

### ELISA

Both blood and the brain (removing the cerebellum) were used for ELISA test with analysis kits. Use 1% pentobarbital sodium (80 mg/kg) for general anesthesia, collect 1 ml of blood from mice through the posterior orbital plexus, and use the serum for subsequent testing, and the serum was used for further test. The brain tissue was homogenized and the sample was prepared according to the manufacture’s instruction. TNF-α, IL-1β, IL-6, and IL-10 concentrations were evaluated following the manufacturer’s guidelines. Serum ACTH, and GC were detected with ELISA as well. The absorption peak occurred at 450 nm.

### Immunohistochemistry

Following animal sacrifice, brain tissues underwent fixation in 4% paraformaldehyde before paraffin embedding. Tissue blocks were sliced into 4 μm sections for further test. The primary and secondary antibodies were respectively incubated with the brain section overnight at 4°C and 50 min at ambient temperature. Following a 10-minute DAPI incubation, microscopy was used for image analysis. (NIKON ECLIPSE C1 with NIKON DS-U3).

### Ultra-high performance liquid chromatography-MS/MS (UPLC-ESI-MS/MS)

After the sample was freeze-dried under vacuum, 1000 μL of extraction solvent (methanol: acetonitrile: water v/v=1:2:1) was added to 50 mg dried sample, and vortexed for 30 s. Following a 10-min ice-water bath sonication, samples underwent centrifugation (4°C, 15 min, 12,000 rpm). Supernatants were then filtered using 0.22 μm membranes. For HPLC study, a reversed-phase HPLC system (UPLC, Waters Acquity I-Class PLUS) with Acquity UPLC HSS T3 column (Waters Corporation, Milford, Massachusetts, 2.1× 100 mm, 1.8 µm) was used. The temperature for the column is 50°C. The sample injection volume is 4 μL. Eluent A contains 0.1% formic acid and 5 mM ammonium acetate, and eluent B is 0.1% formic acid (v/v) in acetonitrile. The flow rate is 0.35 mL/min. The start gradient for elution was 98% A and 2% B, and the gradient was maintained for 1.5 min. Then the gradient was linearly set up to reach 50% A with 50% B in the next 5 min, and 2% A with 98% B in 9 min. The gradient was maintained for 1 min then switched to 98% A and 2% in 1 min and maintained for 3 min. In MS analysis (Applied Biosystems QTRAP 6500+), dual ionization modes were utilized with multiple reaction monitoring (MRM) detection. The mass spectrometry source was configured with the following parameters: the curtain gas (CUR) was maintained at 35, while the collision gas (CAD) was set to medium. The ion spray voltage (IS) was adjusted to 5500 volts for positive ions and -4500 volts for negative ions. The temperature (TEM) was kept at 550 degrees, with ion source gas 1 (GS1) and ion source gas 2 (GS2) set to 50 and 55, respectively. Data acquisition and processing were carried out using Analyst software version 1.6.2 (ABSciex, Foster City, CA, USA).

### Statistical analysis

The data exhibited a normal distribution with equal variance across the board. We ran a one-way ANOVA, and to pinpoint exactly where the differences lay, we followed it up with a Least Significant Difference (LSD) post-hoc test. Any p-value dipping below the 0.05 mark was flagged as statistically significant. The number crunching was all done in SPSS 26.0 (IBM SPSS Statistics, Chicago, IL, USA).

## Results

### PNF ameliorates the depression like behaviors of CUMS mice 

**Figure 1:**
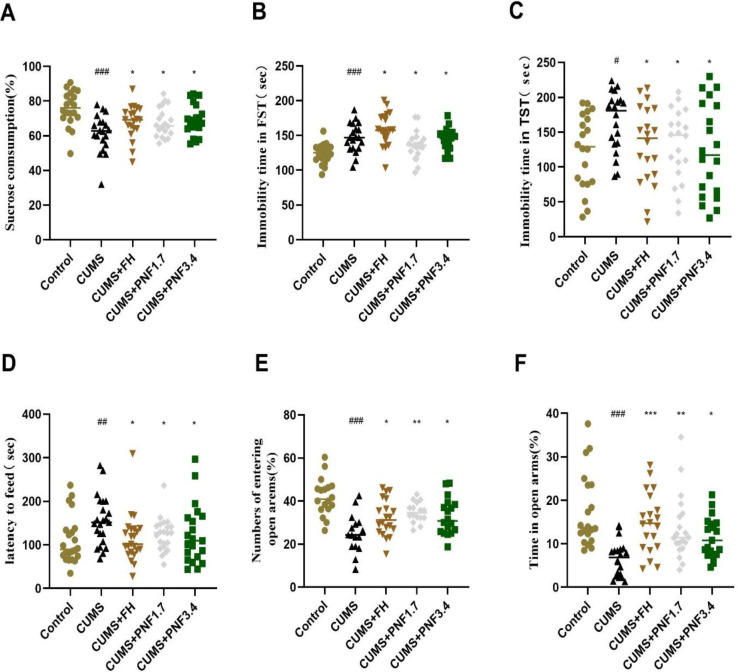
PFN ameliorates the depression-like behaviors in CUMS mice. (A) Sucrose consumption in sucrose preference test. (B) Immobility time in forced swimming test (FST). (C) Immobility time in tail suspension test (TST). (D) The latency to feed in open-field test. (E) The numbers the mice entering the open arms in elevated plus maze test. (F) The time the mice stay in the open arms in elevated plus maze test. The data are presented as the mean ± standard deviation. #p<0.05，##p<0.01, and ###p<0.001, vs CON group; *p<0.05, **p<0.01, and ***p<0.001, vs CUMS group. PFN: Panax notoginseng flower; FH: fluoxetine hydrochloride.

As showed in Figure 1, the sucrose consumption of CUMS mice dropped markedly compared to the control group (p<0.01). Administration of PNF 1.7 g/kg and 3.4 g/kg significantly attenuated the CUMS-induced sucrose intake reduction (p<0.05). In forced swimming test and tail suspension test, the immobility time significantly increased after CUMS insults (vs control, p<0.01, p<0.05 respectively), which were markedly prolonged after PNF treatment (vs CUMS, p<0.05). In open-field test, food exploring latency was significantly prolonged after CUMS (vs control, p<0.01), and this time was significantly shortened after PNF treatment (vs CUMS, p<0.05). Finally, in the elevated plus maze test, the time spending in open arms and the numbers entering the open arms were all markedly reduced in CUMS mice (vs control, p<0.01), and both significantly increased after PNF treatment (vs CUMS, p<0.01). All above results suggest that PNF, both 1.7 g/kg and 3.4 g/kg, alleviate the depressive-like behaviors in murine models caused by CUMS insults. 

### PNF alleviates the systemic and neuronal inflammation in CUMS mice

**Figure 2:**
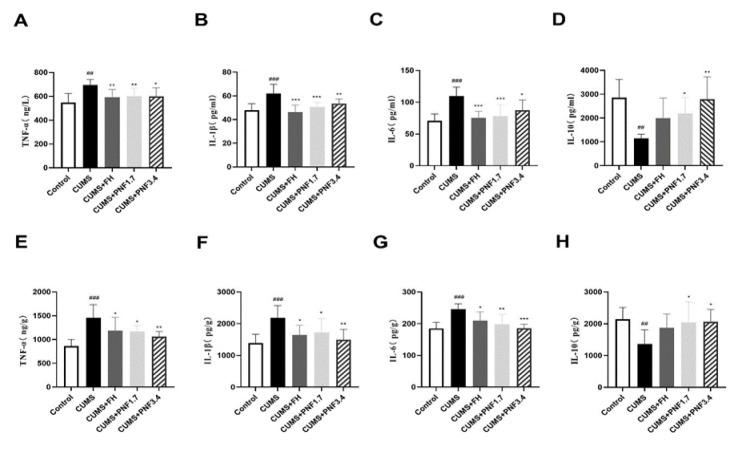
The effects of PNF on systemic inflammation and neuroinflammation in CUMS mice. (A-D) The concentration of IL-1β, IL-6, TNF-α, and IL-10 in serum. (E-H) The content of IL-1β, IL-6, TNF-α, and IL-10 in the brain**.** The data are presented as the mean ± standard deviation. #p<0.05，##p<0.01, and ###p<0.001, vs CON group; *p<0.05, **p<0.01, and ***p<0.001, vs CUMS group. PFN: Panax notoginseng flower; FH: fluoxetine hydrochloride. N=6.

Systemic and neuronal inflammation was analyzed by monitoring the typical inflammatory molecules in serum and brain respectively. Figure 2 showed that in serum CUMS induced a significant increase of TNF-α, IL-1β, and IL-6, whereas a significant reduction of IL-10 (vs control, p<0.01). Following PNF therapy, the elevation of serum TNF-α, IL-1β, and IL-6 induced by CUMS were significantly suppressed (vs CUMS, TNF-α: both p<0.05; IL-1β: p<0.01; IL-6: p<0.01 for 1.7 g/kg, p<0.05 for 3.4 g/kg), and the reduction of serum IL-10 by CUMS were restored significantly (vs CUMS, p<0.05 for 1.7 g/kg, p<0.01 for 3.4 g/kg). In the brain, notable elevations in TNF-α, IL-1β, and IL-6 were also observed (vs control, p<0.01), and a marked decrease of IL-10 was detected (vs control, p<0.01) in CUMS mice. When treated with PNF, the surge of TNF-α, IL-1β, and IL-6 in the brain induced by CUMS were significantly inhibited (vs CUMS, TNF-α and IL-1β: p<0.05 for 1.7 g/kg, p<0.01 for 3.4 g/kg; IL-6: p<0.01 for both), and the reduction of IL-10 by CUMS were restored (vs CUMS, p<0.05 for both).

### PNF inhibits the HPA activation induced by CUMS in mice

**Figure 3:**
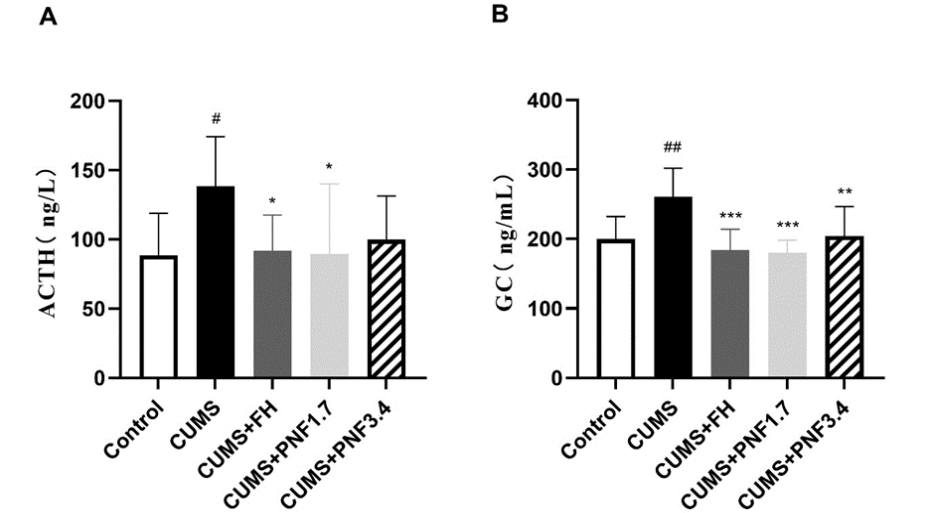
PNF inhibits the HPA activation induced by CUMS in mice. (A) The serum levels of ACTH in CUMS mice. (B) The serum level of glucocorticoids in CUMS mice. The data are presented as the mean ± standard deviation. # p<0.05，##p<0.01, and ###p<0.001, vs CON group; *p<0.05, **p<0.01, and ***p<0.001, vs CUMS group. PNF: panax notoginseng flower; FH: fluoxetine hydrochloride. n=6

ACTH and glucocorticoids are the core molecules in the hypothalamus-pituitary-adrenocortical system (HPA). The results showed that after CUMS insults, the serum ACTH and glucocorticoids were significantly increased (vs control, p<0.05 for ACTH and p<0.01 for glucocorticoids), indicating an activation of HPA after CUMS insults. Treatment with PNF obviously attenuated the elevation of serum ACTH and glucocorticoids induced by CUMS (vs CUMS, ACTH: p<0.05 for 1.7 g/kg, p=0.058 for 3.4 g/kg; glucocorticoids: p<0.01 for both), Figure 3.

### PNF suppresses microglial and astrocytes activation in the brain of CUMS mice

**Figure 4:**
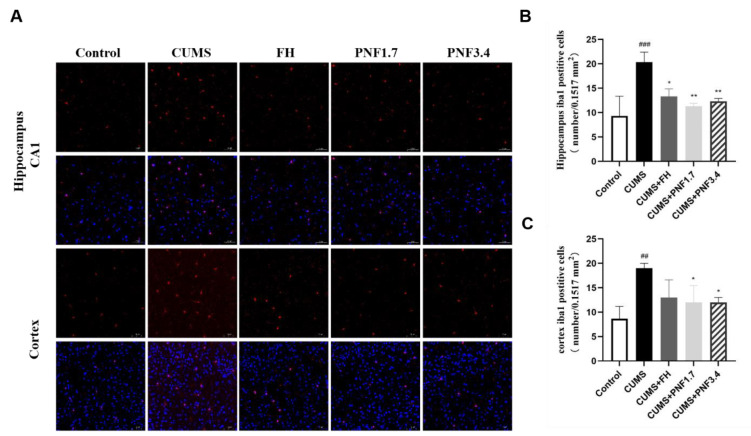
The effects of PNF on microglia activation in the brain of CUMS mice. (A) Microglia in hippocampus and cerebral cortex of the brain in CUMS mice. (B) Iba-1 positive cells per 0.1517 mm^2^ in hippocampal CA1 region. (C) Iba-1 positive cells per 0.1517 mm^2^ in cortex. The data are presented as the mean ± standard deviation. # p<0.05，## p<0.01, ### p<0.001, vs CON group; * p<0.05, ** p<0.01, and *** p<0.001, vs CUMS group. PFN: Panax notoginseng flower; FH: fluoxetine hydrochloride. n=4

**Figure 5:**
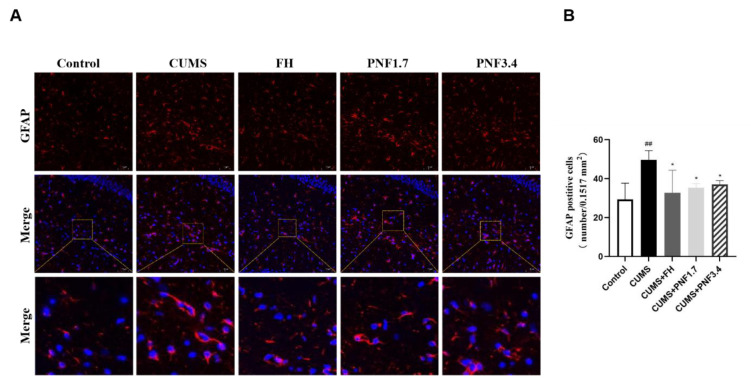
The effects of PNF on astrocytes in the brain of CUMS mice. (A) Activation of astrocytes in the brain of CUMS mice. (B) GFAP positive cells per 0.1517 mm^2^. The data are presented as the mean ± standard deviation. # p<0.05，## p<0.01, ### p<0.001, vs CON group; * p<0.05, ** p<0.01, and *** p<0.001, vs CUMS group. PFN: Panax notoginseng flower; FH: fluoxetine hydrochloride. n=4

It is believed that an overactive presence of microglia and astrocytes plays a role, whether directly or indirectly, in neuroinflammation. To get a better handle on how proprioceptive neuromuscular facilitation (PNF) affects things after chronic unpredictable mild stress (CUMS), we took a look at the activation of microglia and astrocytes-indicated by Iba-1 and GFAP positive cells in the cerebral cortex and hippocampus regions. The findings revealed a marked rise in Iba-1 and GFAP positive cells in the brain of CUMS group mice significantly increased (vs control, Iba-1: p<0.01 in both hippocampus and cortex; GFAP: p<0.01). As shown in Figure 4 and Figure 5, CUMS triggered significant microglial activation characterized by enlarged cell soma and thicker extensions in both observed areas, and astrocytes were obviously activated after CUMS insults as well. When treated with PNF, both for 1.7 g/kg and 3.4 g/kg, the activation of microglia and astrocytes in the brain were significantly inhibited (vs CUMS, Iba-1 in hippocampus: p<0.01 for both; Iba-1 in cortex: p<0.05 for both; GFAP: p<0.05 for both). 

### Chemical components in PNF

**Figure 6:**
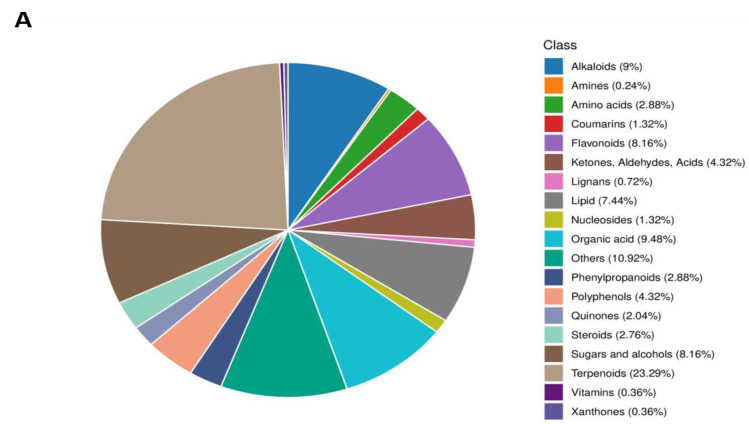
Main components and main ginsenosides in PNF. (A) Proportion of primary components in PNF. PFN: Panax notoginseng flower

As determined by UPLC-ESI-MS/MS, the major constituents of PNF are terpenoids (23%), organic acids (9.5%), alkaloids (9%), and flavonoids (8.2%), showed in Figure 6 A. In terpenoids, Ginsenosides are the major active ingredients, in which Ginsenoside Rh1 and Ginsenoside F1 are the main components, showed in Table 1.

**Table 1:** The proportion of main ginsenosides in PNF.

**Analyte**	**Proportion in triterpenoids（%）**	**Proportion in PNF（%）**
Ginsenoside Rb2	0.0755806	0.0176027
Koryoginsenoside R1	0.0093490	0.0021774
Ginsenoside Rk1	0.0023156	0.0005393
Ginsenoside Rd	0.0105231	0.0024508
Ginsenoside Rb1	0.0407240	0.0094846
Ginsenoside F5	0.0281124	0.0065474
Ginsenoside F3	0.0000064	0.0000015
Ginsenoside Ro	0.0145311	0.0033843
Ginsenoside Rh1	4.8060475	1.1193285
Ginsenoside F1	1.8462147	0.4299834
20(S)-Ginsenoside Rg3	0.2687394	0.0625894

## Discussion

Our research revealed that PNF effectively alleviates depression-like symptoms in mice triggered by CUMS. Following PNF administration, a notable decrease in pro-inflammatory cytokines was observed in both the serum and brain tissues of the CUMS-exposed mice. The elevation of ACTH and GS in serum induced by CUMS were also suppressed by PNF treatment. Further studies suggest that both astrocytes and microglia activation in the brains of CUMS mice were obviously attenuated by PNF treatment. This study initially documents PNF's positive impact on depression-like behaviors. 

*Panax notoginseng* flower is the flower buds of *P. notoginseng* (Burk.). Like the medicinal materials of Ginseng, PNF is also rich in ginsenosides, such as ginsenoside Rh1 and ginsenosides F, etc. as detected in UPLC/MS study. A number of studies suggested that ginseng can ameliorate the depression-like behaviors in animal models (Liu et al. 2016; Wang et al. 2017; Kim et al. 2010; Chen et al. 2017), but there are no comprehensive studies have been conducted on PNF against depression. In this study, using the CUMS depression model, through multiple behavioral modality tests, it was showed that PNF had significant improvement effects on CUMS-related depressive symptoms in murine models, with effects similar to those of the standard antidepressant medication, fluoxetine, which was also tested in this study. The results shed a light to the treatment of depression in the future.

Inflammatory molecules, such as cytokines, are elevated in the blood of depressed patients, suggesting a correlation between inflammation and depression. In this research we observed that CUMS notably elevated inflammatory cytokines in the blood of mice, suggesting that the inflammatory response induced by CUMS is systemic. In addition to cytokines, the ACTH and glucocorticoids in the blood were also elevated after CUMS insults. Giving glucocorticoids is usually considered to have an inhibitory effect on the inflammatory response, our findings in this study suggest that the inflammatory response and HPA were in an unmatched scenario after CUMS insults. Further studies showed that the inflammatory factors in brain of CUMS mice were markedly elevated, indicating that the inflammatory response of the central nervous system was obvious. After PNF treatment, the inflammatory cytokines both in the blood and brain of CUMS mice significantly decreased. At the same time, ACTH and glucocorticoids in the blood were also significantly reduced by PNF treatment, suggesting that the overall inflammation caused by CUMS was significantly alleviated by PNF intervention.

Microglia are thought to be resident macrophages in the central nervous system and are key regulators of the brain's immune microenvironment (Nayak et al. 2014). Once activated, microglia secrete a variety of inflammatory mediators that can promote a neuroinflammatory response in the brain, potentially causing significant alterations in neurologic function (Réus et al. 2015). In recent years, it has been found that astrocytes in the brain are also involved in inflammatory responses (Colombo and Farina 2016). In this study, we observed that CUMS stimulation in mice significantly activated microglia and astrocytes in the brain, which was characterized by massive increase of Iba1- and GFAP-positive cells, as well as significant changes in cell morphology. After PNF treatment, the activation of these two types of glial cells in the brains of CUMS mice was markedly suppressed, suggesting that the inflammation in the brain was significantly alleviated.

Ginsenosides are the main active ingredients of ginseng. Numerous studies have shown that ginsenosides impact the neural network in diverse ways, including anti-inflammatory (Jang et al. 2023), antioxidant (Saw et al. 2012), nerve regeneration promotion (Wang et al. 2015), and anti-apoptosis (Hu et al. 2020). It is suggested that ginsenosides can ameliorate a variety of neurological and psychiatric disorders. In this study, we found that the extract of PNF contains various compounds such as ginsenosides and flavonoids, etc. Although we cannot attribute the antidepressant effect of *P. notoginseng* flower solely to ginsenosides, the importance of ginsenosides should be considered with priority. Our previous study found that ginsenosides alleviate neuroinflammation and improve depression and anxiety-like behaviors caused by various insults (Zhang et al. 2021; Xie et al. 2024) . It is therefore reasonable to speculate that alleviation of neuroinflammation is closely related with the antidepressant effects of PNF, and ginsenosides may be the key players among other ingredients. 

In summary, this study demonstrated PNF's antidepressant impact on CUMS-induced depressive mice, and preliminarily explored its chemical composition and the mechanism of action. This research offers promising prospects for future depression therapies.
